# Geographical Differences in SARS-CoV-2 Antibody Response Dynamics and Neutralisation Profiles to Mild COVID-19: Lessons from a UK–Uganda Comparison

**DOI:** 10.3390/vaccines13040336

**Published:** 2025-03-21

**Authors:** Laban Kato, Jackson Sembera, Gerald Kevin Oluka, Joseph Ssebwana Katende, Juliana Bemanzi, Violet Ankunda, Peter Ejou, Ashwini Kurshan, Carl Graham, Jeffrey Seow, Katie J. Doores, Michael H. Malim, Julie M. Fox, Pontiano Kaleebu, Jennifer Serwanga

**Affiliations:** 1 Viral Pathogens Research Theme, Medical Research Council, Uganda Virus Research Institute and London School of Hygiene & Tropical Medicine (MRC/UVRI & LSHTM) Research Unit, Entebbe 49, Uganda; 2 Department of Immunology, Uganda Virus Research Institute, Entebbe 49, Uganda; 3 Department of Infectious Diseases, School of Immunology & Microbial Sciences, King’s College London, London SE1 9RT, UK

**Keywords:** SARS-CoV-2, COVID-19, geographical variations, neutralising antibodies, UK, Uganda, spike, nucleoprotein, IgG, IgM

## Abstract

**Background**: The global SARS-CoV-2 pandemic revealed stark variability in clinical outcomes across populations, underscoring the need for region-tailored vaccination strategies. To inform standardised global immunisation efforts, this study compared longitudinal binding antibody responses and neutralisation capacities in mild COVID-19 cases from Uganda and the United Kingdom (UK). **Methods**: IgG responses to spike (S) and nucleocapsid (N) proteins, along with IgM responses to S and receptor-binding domain (RBD) proteins, were assessed in 29 Ugandan and 14 UK participants over 84 and 82 days, respectively. Antibody levels were quantified using a validated enzyme-linked immunosorbent assay (ELISA), alongside pseudovirus neutralisation assays targeting the D614G variant. **Results**: Ugandan participants exhibited higher early IgG and IgM levels, particularly against spike and RBD, with a rapid onset of responses that waned quickly. UK participants showed a slower but sustained increase in IgG and IgM levels. Neutralisation titres revealed elevated responses in 16.4% of Ugandan participants (>2000) compared to 4.5% of UK participants, suggesting a greater sensitivity to viral neutralisation. Conversely, 31.8% of UK participants exhibited low titres (<25) compared to 14.8% of Ugandan participants, indicating differences in resistance mechanisms. Neutralisation correlated strongly with spike and receptor-binding domain IgG in the UK cohort but showed weaker correlations in Ugandan participants. **Conclusions**: These findings highlight distinct population-level immune responses, suggesting that geographic factors shaped the quality and durability of SARS-CoV-2 immunity. Tailored vaccination strategies are essential to optimise immunity across diverse populations and improve global epidemic preparedness.

## 1. Introduction

The global outbreak of SARS-CoV-2 resulted in a wide spectrum of COVID-19 clinical outcomes, ranging from asymptomatic infections to severe, life-threatening disease requiring intensive care [1,2,3]. Understanding the immune response to SARS-CoV-2 in mild cases is crucial for shaping public health interventions and optimising vaccine strategies [4,5,6], with immune profiling providing essential insights in these efforts. Geographical [6,7,8,9] and demographic factors, including age, gender, and regional differences [10,11,12], influence virus-specific immune responses, including those to SARS-CoV-2, with older adults and males exhibiting lower antibody levels. These variations may influence the magnitude and durability of antibody responses, which are key indicators of immune protection [13], and highlight the role of geographical factors in shaping immune responses to SARS-CoV-2 across diverse populations.

This study compared the immune responses of mild COVID-19 cases from the United Kingdom and Uganda, focusing on the longitudinal dynamics of key immunoglobulins, including spike IgG, nucleocapsid IgG, spike IgM, nucleocapsid IgM, and receptor-binding domain (RBD) IgM. These domains were prioritised as distinct aspects of the host’s adaptive immune response, each with critical implications for clinical outcomes and vaccine effectiveness. Spike-directed IgG, recognized for its durable response, is essential for neutralizing activity and is often associated with long-term immunity [14], contributing to reduced susceptibility to severe reinfection. Nucleocapsid IgG offers insights into immunity generated by natural infection, which may vary geographically due to differences in viral exposure. Early IgM responses, including spike and nucleocapsid IgM, play a crucial role in the initial immune defence, correlating with rapid viral clearance, and potentially influencing clinical outcomes. RBD-specific IgM, which targets the virus’s receptor-binding interface, is essential for blocking viral entry and contributes to an immediate, potent immune response. By examining these immunoglobulin profiles across distinct populations, this study aimed to elucidate immune markers that not only signify robust natural defence but also inform the design of highly effective vaccines tailored to produce rapid, sustained, and protective immunity. We aimed to uncover commonalities and differences in immune kinetics between these populations to deepen our understanding of how regional and demographic factors influence SARS-CoV-2 immune responses.

## 2. Materials and Methods

### 2.1. Study Population

The Ugandan cohort was recruited during the first wave of the pandemic, with samples collected within a clearly defined time window. Participants were identified as acute-phase PCR-positive cases (within 7 days of initial SARS-CoV-2 infection) through mandatory border screening protocols for all incoming and outgoing truck drivers. Most exhibited very mild or asymptomatic presentations, as previously described [15], ensuring a representative sample for studying early infection dynamics in a high-mobility population. The Ugandan cohort was comprised exclusively of participants with these infection profiles, since all of the SARS-CoV-2 cases isolated to this study’s site were mild or asymptomatic. Although the study was not initially designed for comparison, we selected post-collection samples that allowed for meaningful comparisons. To ensure comparability, the UK cohort was matched to include only individuals with mild or asymptomatic SARS-CoV-2 infections. The UK cohort was recruited based on a defined timeline post-onset of symptoms. Participants were chosen based on confirmed mild SARS-CoV-2 infection, aligned with the World Health Organisation’s classification for mild cases [1]. Consequently, we included 14 individuals from the United Kingdom and 29 from Uganda, collected early in the pandemic, between 2 July 2020 and 7 July 2021. At the time of sample collection, the predominant variant waves in Uganda were the A.23.1 and B.1.617.2 variants [16,17,18], while, in the UK, it was the B.1.1.7 and B.1.351 variants [19,20,21]. The Ugandan cohort eligibility criteria necessitated rt-PCR-confirmed COVID-19, the lack of severe symptoms, and the availability of longitudinal serum samples for immunological assessment, while the UK cohort was collected based on days post-onset of symptoms. Individuals with immunocompromising conditions were excluded to maintain comparability among the datasets. All of the participants in this study were unvaccinated at the time of sample collection. The median age of the Ugandan cohort was 33 years (IQR: 28–37), with a majority male representation (69%). Conversely, the UK cohort exhibited a median age of 45.5 years (IQR: 25.5–67.3) and a balanced male-to-female ratio of 50% (Table 1). Ethical approval for the study conducted in Uganda was obtained from the Uganda Virus Research Institute Research Ethics Committee (UVRI REC; reference GC/127/833) and the Uganda National Council for Science and Technology (UNCST; reference HS637ES). For the Ugandan cohort, serum samples were collected following informed written consent from the participants. In the UK cohort, surplus serum samples were retrieved from the routine biochemistry laboratory at St. Thomas’ Hospital, London, UK, at the point of discard. The samples were anonymised and linked to a limited clinical dataset by the direct care team under an established ethics framework (REC reference 18/NW/0584), with expedited approval.

### 2.2. Study Design

In the Ugandan cohort, blood samples were collected from individuals at various intervals: weekly for the first month post-diagnosis, then monthly for three months, and subsequently at quarterly intervals for up to one year. A parallel cohort in the UK was selected for analysis based on similar sampling schedules. Samples from the Ugandan cohort were processed and analysed at the Uganda Virus Research Institute (UVRI), while UK samples were analysed locally in the UK. Both sites conducted analyses independently, adhering to standardised protocols to ensure contextual relevance and methodological consistency.

Serum was separated for the quantitative assessment of antibody responses, focusing on the following key markers: spike (S) IgG, nucleocapsid (N) IgG, spike IgM, nucleocapsid IgM, and receptor-binding domain (RBD) IgM. Antibody levels were measured using a conventional enzyme-linked immunosorbent assay (ELISA), with the assay protocol independently optimised for the Ugandan [22] and the UK populations [23] to ensure accuracy and sensitivity for each population.

### 2.3. Validated Enzyme-Linked Immunosorbent Assay (ELISA)-Based Quantification of SARS-CoV-2-Specific IgG, IgM, and IgA Antibodies

We used a validated in-house ELISA [22,23] to quantify the SARS-CoV-2-specific IgG, IgM, and IgA antibodies, targeting the spike and nucleocapsid, which are the key markers of SARS-CoV-2 immunity. ELISA plates (Greiner Bio-One, #655001, Kremsmünster, Austria) were coated with 3 μg/mL of spike or nucleocapsid antigen (R&D Systems, #10474-CV-01M, #10474-CV-01M, Minneapolis, MN, USA), optimised to achieve maximum sensitivity and specificity. Antibody concentrations were measuring as optical density (OD) values at 450 nm and converted to nanograms per millilitre (ng/mL) using a standard curve generated from serial dilutions of known antibody concentrations. The anti-spike monoclonal antibody CR3022 and the anti-nucleoprotein mAb CR3009 (BEI Resources, Manassas, VA, USA) were used as positive controls, along with the WHO-designated WHO 20/136 and an internal donor sample positive and negative control.

We established seropositivity thresholds using receiver operating characteristic (ROC) analysis, maximising the sensitivity and specificity to distinguish true positive and negative responses. Positive controls consisted of longitudinal samples collected at peak antibody titres from individuals with PCR-confirmed SARS-CoV-2 infection during primary infection, while pre-pandemic samples collected over 10 years prior were used as negative controls. Seropositivity thresholds were 0.432 for IgG, 0.459 for IgM, and 0.226 for IgA against the spike protein, and 0.454 for IgG, 0.229 for IgM, and 0.225 for IgA against the nucleocapsid protein [22]. We validated these thresholds using the World Health Organisation’s (WHO) anti-SARS-CoV-2 verification standards panel (WHO 20/B770-02 S-IgG), achieving 100% sensitivity and 100% specificity. Given the limited availability of WHO reference materials, we derived binding antibody units (BAU/mL) through a validated conversion model, facilitating comparison across studies. The primary data are presented in ng/mL, with BAU/mL values provided for standardisation.

### 2.4. Assessment of Neutralising Antibody Titres Using SARS-CoV-2 Pseudotyped Viruses

To investigate neutralising antibody responses against SARS-CoV-2, we generated pseudotyped viruses incorporating the D614G spike protein variant using an HIV-based lentiviral pseudotyping system [24]. HEK-293T cells (4.0 × 10^6^) were seeded into 10 mL of complete Dulbecco’s Modified Eagle’s Medium (DMEM), supplemented with 10% foetal bovine serum (FBS) and 1% penicillin–streptomycin (Pen/Strep). The cells were incubated at 37 °C in a humidified 5% CO_2_ atmosphere for 20 h. After replacing the media with fresh DMEM growth media, we co-transfected the cells with 100 μg of PEI-Max (1 mg/mL) (PEI-Max, Polysciences, PA, USA) and plasmids encoding HIV-Luc (15 μg), HIV gag/pol (10 μg), and the SARS-CoV-2 D614G spike protein (5 μg). Following a 48-h incubation under identical conditions, we harvested the viral supernatant, filtered it through a 0.45 μm membrane, and aliquoted the material. Pseudovirus titres were determined, and aliquots were stored at −80 °C until use.

For the neutralisation assays, we heat-inactivated plasma samples at 56 °C for 30 min and serially diluted them in three-fold increments in DMEM. Diluted plasma samples were pre-incubated with the pseudotyped virus for 1 h at 37 °C in flat-bottom 96-well plates along with internal positive control sera and monoclonal antibodies. Afterwards, 10,000 HeLa cells stably expressing the human ACE-2 receptors were added to each well. The plates were incubated at 37 °C in a humidified 5% CO_2_ environment for 72 h. Luciferase activity, indicative of viral entry, was quantified using the Bright-Glo luciferase reagent (Promega, Madison, WI, USA) on a Victor™ X3 multilabel plate reader (Perkin Elmer, Waltham, MA, USA). Neutralising antibody titres were calculated as ID_50_ values, defined as the reciprocal plasma dilution achieving a 50% inhibition of the luciferase activity relative to virus-only controls. The ID_50_ values were determined using non-linear regression and curve-fitting with the drc package in R software v4.2.2.

### 2.5. Statistical Methods

We conducted a comparative analysis of longitudinal immune responses in mild COVID-19 cases from UK and Ugandan cohorts. The primary objective was to assess differences in antibody dynamics and neutralisation profiles over time between the two populations. The demographic characteristics of study participants were summarised using descriptive statistics. Continuous variables were presented as medians with interquartile ranges (IQRs), while categorical variables were reported as frequencies and percentages. To examine the temporal dynamics of the antibody responses, we applied locally weighted scatterplot smoothing (LOWESS) to visualise the trends for key immunoglobulin isotypes, including spike IgG, nucleocapsid IgG, spike IgM, nucleocapsid IgM, and RBD IgM. We assessed the differences in the neutralisation titres between the cohorts using the Wilcoxon rank-sum test for unpaired comparisons, a non-parametric approach suitable for small or skewed data distributions. Statistical significance was defined as a *p*-value of ≤0.05. All of the analyses and data visualisations were performed in R Version 4.2.2.

## 3. Results

### 3.1. Antibody Kinetics Revealed Divergent Patterns Between Ugandan and UK Cohorts

**Figure 1:**
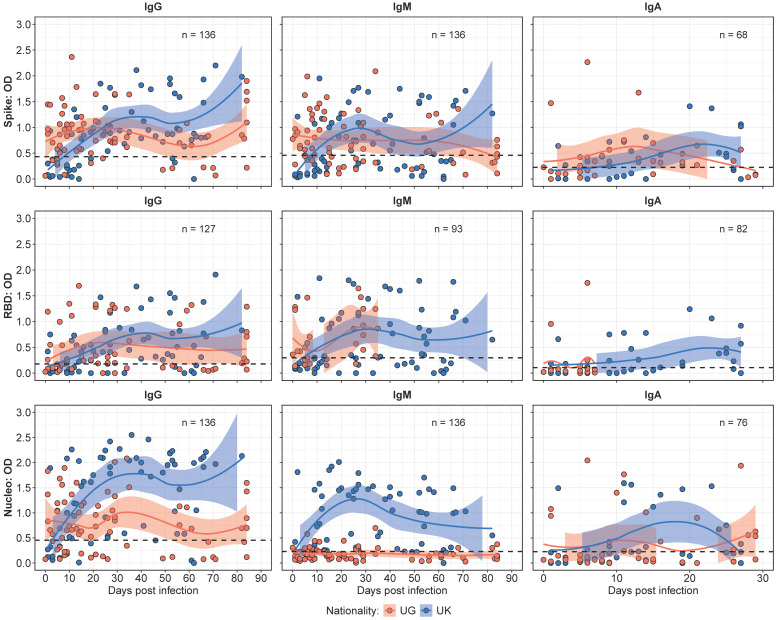
Comparative binding antibody kinetics between the Ugandan (UG) and UK cohorts over time. Figure 1 presents LOWESS curves illustrating the temporal dynamics of the IgG, IgM, and IgA antibody responses in the UG and UK cohorts. The IgG responses initially peak higher in the UG cohort, but are later surpassed by the UK cohort, maintaining elevated levels beyond day 20. Similarly, the IgM responses exhibit a pronounced increase in the UK cohort over time, while the UG cohort shows negligible N-IgM responses. The IgA antibody responses demonstrate an initial peak in the UG cohort, with the UK cohort progressively surpassing these levels within the 30-day observation period.

The binding antibody kinetics, depicted in Figure 1, demonstrated distinct patterns between the Ugandan (UG) and UK cohorts over a 90-day observation period. Median IgG responses were initially higher in the UG cohort (median OD 0.88; IQR: 0.53–1.20) compared to the UK cohort (median OD 0.19; IQR: 0.07–0.36). However, IgG responses in the UK cohort surpassed those in the Ugandan cohort by day 20, and remained higher for the rest of the study.

IgM responses exhibited a similar trend, with levels that remained stable throughout the study. The UG cohort demonstrated higher median IgM levels early in the study period. In contrast, the UK cohort displayed a greater increase over time, leading to significantly higher levels by the mid-point of the study (*p* < 0.05). Notably, the Ugandan cohort lacked detectable N-IgM responses throughout the study period, while the UK cohort showed steadily increasing N-IgM levels over time, underscoring a key antigenic distinction between the cohorts. IgA responses assessed over the 30 days exhibited higher median IgA levels at baseline; however, the UK cohort displayed a progressive increase that surpassed UG levels by day 20.

Previously [15], we determined the threshold for the use of N-IgG fold changes after the initial infection peak (~35 days) to track the reinfection status of the study participants. In this study, we found three participants (1 UK, 2 UG) who had a greater than 2-fold increase in N-IgG antibody levels after the initial peak at 30–35 days. In the UK participant, a 16-fold increase in N-IgG at 44 days post-infection was observed, while one participant from the UG cohort had a 4-fold increase in N-IgG at 82 days post-infection and the other had a 2-fold increase in N-IgG levels at 68 days post-infection. This finding highlights a negligible effect of reinfections in the overall observed immune response profiles in this study.

### 3.2. Neutralisation Titres Against the D614G Variant Reveal Regional Differences in Sensitivity and Resistance

**Figure 2:**
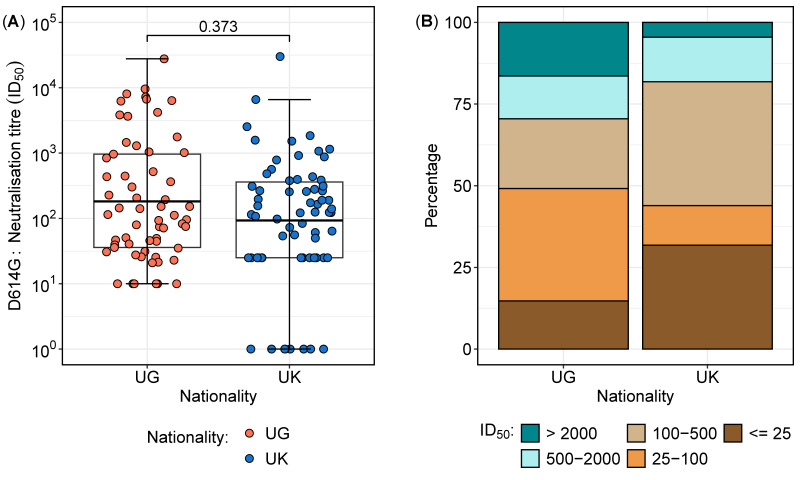
Neutralisation titre distribution and proportions between the Ugandan and UK cohorts. Figure 2 illustrates the neutralisation titre profiles between the Ugandan (UG) and UK cohorts. (**A**) presents boxplots comparing the geometric mean titres (GMTs) and interquartile ranges of the neutralisation titres, demonstrating no significant difference in the overall distributions between the two cohorts. (**B**) highlights the proportions of subjects across varying neutralisation titre ranges, revealing a higher percentage of Ugandan subjects with titres exceeding 2000, indicative of enhanced sensitivity, while the UK cohort displays a greater proportion of subjects with titres below 25, reflecting increased resistance.

Overall, a total of 127 samples were analysed from 41 participants, comprising 27 from Uganda and 14 from the UK. The distribution of neutralisation titres against the D614G variant varied between the two cohorts (Table 2 and Figure 2). The overall geometric mean titres (GMTs) and interquartile ranges revealed that the largest proportion of samples (29.9%) had neutralisation titres between 100 and 500 (Table 2), with no statistically significant difference between the two cohorts (*p* = 0.373) (Figure 2A). However, a deeper analysis of the distributions highlighted notable differences in the proportions of subjects across neutralisation ranges (Figure 2B). Notably, the Ugandan participants exhibited a significantly higher proportion of high neutralisation titres (>2000) compared to their UK counterparts (16.9% vs. 4.5%; *p* = 0.0282, proportion test), indicating a stronger neutralising antibody response in the Ugandan cohort. In contrast, 31.8% of the UK participants had titres below 25 compared to 14.8% in the Ugandan population (*p* = 0.0200, proportion test), suggesting a weaker neutralisation response in the UK group. These findings suggest distinct immune response patterns between the cohorts, potentially influenced by differential antigenic exposures or immunogenetic factors.

**Figure 3:**
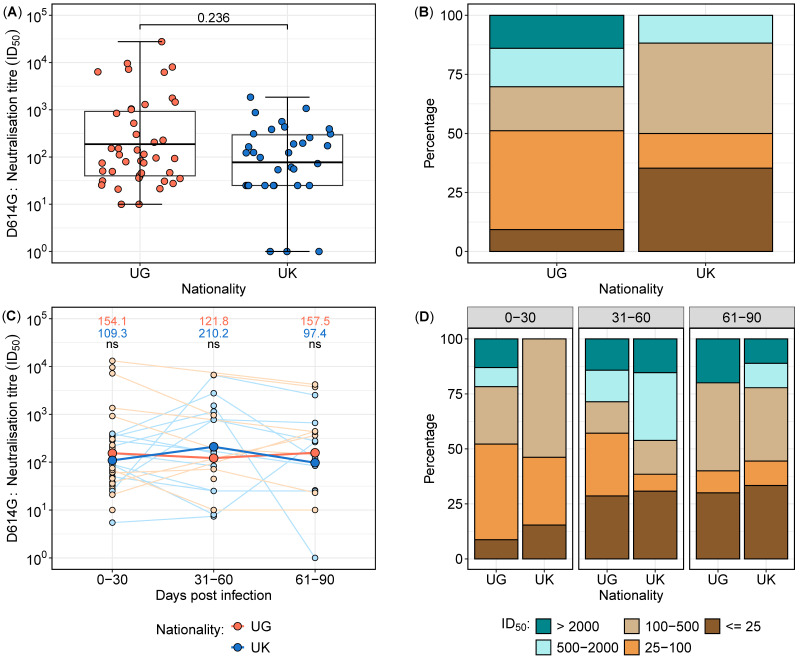
Longitudinal neutralisation titres in the UK and UG cohorts over time, stratified by time intervals from 0 to 90 days post-infection. Boxplots depicting the distribution of neutralisation titres in samples collected within the first 30 days post-infection from UK and UG populations (**A**). Proportional distribution of neutralisation titres in the first 30 days, categorised into low, moderate, and high titres, comparing the UK and UG cohorts (**B**). Longitudinal neutralisation titres stratified into three time intervals (0–30, 31–60, and 61–90 days post-infection), presented as a line graph, with geometric mean titres displayed at each time point (**C**) Red lines and data points represent Ugandan participants while blue lines and data points represent UK participants. ns means not statistically significant. Proportional distribution of neutralisation titres across the three time intervals, categorised into low, moderate, and high titres, illustrating the temporal variation between the UK and UG populations (**D**).

Considering the neutralisation data points within the first 30 days, neutralisation responses were assessed in 77 samples collected from 36 subjects (Figure 3). Neutralisation titres exhibited higher geometric mean levels in the Ugandan cohort compared to the UK cohort (Figure 3A); however, the difference was not statistically significant (*p* = 0.236). Stratification by titre categories revealed a greater proportion of low neutralisation titres (<25) in the UK cohort (35.3%) compared to the Ugandan cohort (9.3%). Conversely, 14% of samples from Uganda exhibited titres exceeding 2000, whereas no UK samples reached this threshold (Figure 3B). These findings indicate potential differences in the early neutralisation capacity between the two populations, warranting further investigation into underlying immunological determinants.

To standardise the analysis of the neutralisation data, each participant was assigned a single representative sample per time category. For participants with multiple samples within a given time group, the geometric mean titre (GMT) was calculated to ensure uniform representation. The neutralisation data were stratified into the following three time intervals: 0–30 days, 31–60 days, and 61–90 days post-infection. A total of 75 samples from 41 participants were analysed, with 36 samples collected between 0 and 30 days, 20 samples between 31 and 60 days, and 19 samples between 61 and 90 days (Figure 3C,D).

Comparative analysis of the longitudinal neutralising antibody titres between the UK and Uganda cohorts revealed dynamic changes over time. In the early phase (0–30 days), the Ugandan participants exhibited marginally higher neutralisation titres compared to their UK counterparts. However, by 31–60 days, the UK participants demonstrated a relative increase in neutralisation titres, surpassing those observed in the Ugandan cohort. In the final time category (61–90 days), the UG participants again displayed higher neutralisation titres (Figure 3C,D). Despite these fluctuations, statistical comparisons at each time point did not reveal significant differences (*p* > 0.05 for all comparisons). These findings suggest temporal variations in the neutralisation kinetics between cohorts, potentially influenced by differences in immune priming, boosting, or antigenic exposure history.

### 3.3. Correlation of Neutralisation Titres with S, RBD, and N-IgG Responses Differed Between Nationalities

**Figure 4:**
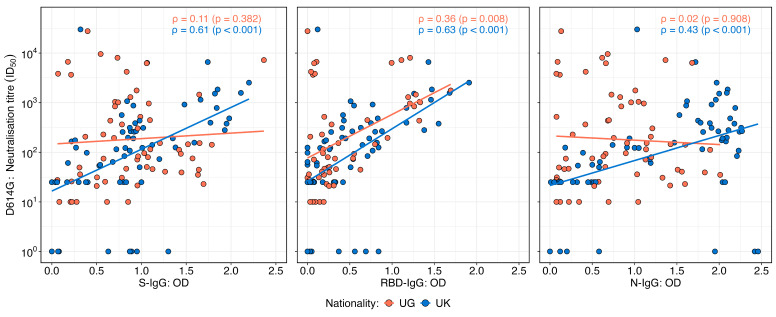
Correlations between neutralisation titres and SARS-CoV-2 D614G spike, RBD, and nucleocapsid IgG binding antibody OD responses stratified by nationality. Figure 4 illustrates the correlations between the SARS-CoV-2 D614G neutralisation titres and IgG optical density (OD) responses targeting spike (S), receptor-binding domain (RBD), and nucleocapsid (N) antigens, stratified by the UK and Uganda populations. Scatter plots and correlation coefficients demonstrate the strength and direction of associations for each antigen-specific response across the two populations. The analysis reveals distinct patterns in the relationship between neutralisation titres and D614G antigen-specific IgG responses by nationality.

Neutralisation titres against the D614G variant exhibited varying correlation patterns with OD levels of IgG targeting the spike, receptor-binding domain (RBD), and nucleocapsid proteins in the UK and Ugandan cohorts (Figure 4). In the UK cohort, neutralisation titres demonstrated a moderate and statistically significant positive correlation with S-IgG (Spearman’s r = 0.61; *p* < 0.001), RBD-IgG (r = 0.63; *p* < 0.001), and N-IgG OD values (r = 0.43; *p* < 0.001), suggesting an association between the binding and functional antibody responses. In contrast, the Ugandan cohort showed no significant correlation between neutralisation titres and S-IgG (r = 0.11; *p* = 0.382) or N-IgG OD values (r = 0.02; *p* = 0.98). However, a weak but statistically significant positive correlation was observed between neutralisation titres and RBD-IgG OD values (r = 0.36; *p* = 0.008). These findings highlight population-specific differences in the relationship between antibody binding and the neutralisation capacity, potentially reflecting variations in immune priming, antigen exposure, or other immunological determinants.

## 4. Discussion

This study provides insights into how regional factors shape humoral immunity to SARS-CoV-2, focusing on mild cases in Uganda and the UK. By comparing longitudinal antibody dynamics between the Ugandan and UK cohorts, our findings reveal crucial differences that can inform vaccine development and public health strategies tailored to diverse populations.

Distinct antibody kinetics between the cohorts underscore key immunological variations. The Ugandan cohort exhibited a rapid and pronounced early antibody response, likely reflective of genetic predispositions, environmental exposures, and prior immunity from endemic pathogens or cross-reactive coronaviruses [25,26,27]. However, this response waned rapidly, suggesting limited durability and underscoring the importance of booster vaccinations in similar populations to sustain long-term protection. In contrast, the UK cohort demonstrated a more gradual but sustained antibody increase, indicative of robust immune memory formation and potential long-term immunity. These divergent kinetics emphasise the necessity of region-specific vaccination strategies to optimise immune responses across diverse populations. These findings align with evidence suggesting that regional variations in immune landscapes can significantly affect the nature and durability of SARS-CoV-2 immunity [28,29].

Neutralisation assays further emphasise these differences, with the Ugandan cohort showing a higher prevalence of individuals with strong neutralising activity against the D614G variant. This suggests a population-specific advantage in rapid viral neutralisation, which may offer immediate short-term protection during outbreaks. In contrast, the UK cohort exhibited a greater proportion of low-titre responders, potentially reflecting reliance on alternative protective mechanisms [30], such as cellular immunity, or non-neutralising antibody functions, which warrant investigations.

Unexpectedly, neutralising antibody titres in the Ugandan cohort did not correlate strongly with binding IgG responses to spike or nucleocapsid proteins. This contrasts starkly with observations in the UK cohort, where strong correlations between these antibody metrics were evident. These observations highlight fundamental differences in immune functionality between populations, prompting questions about the factors shaping neutralising responses in distinct geographical and epidemiological contexts. A possible explanation lies in Uganda’s early exposure to the A.23.1 variant of SARS-CoV-2, which may have influenced the antigenic landscape and shaped the immune response differently. Our past studies showed that Ugandan individuals exposed to A.23.1 exhibited stronger binding and neutralising responses to A.23.1 than to D614G, suggesting that early antigenic priming with A.23.1 might have shaped the regional immune landscape [16]. This could have influenced the specificity and functional relevance of neutralising antibodies, potentially explaining the weaker correlation with generic spike or nucleocapsid IgG responses. These findings suggest that regional immune landscapes may influence not only the quality of antibody responses but also their functional relevance in providing protection against SARS-CoV-2.

The implications of these findings extend beyond SARS-CoV-2, shedding light on the broader interplay between geography, immunity, and pathogen evolution. Tailored vaccination strategies informed by population-specific immune profiles could significantly enhance global epidemic preparedness and response efforts. Additionally, the identification of unique immune response patterns offers a valuable framework for designing vaccines that elicit durable and protective immunity across diverse populations.

This study also provides insights into the potential mechanisms underpinning the observed differences. Ugandan participants’ stronger early responses may reflect a heightened basal immune activation due to endemic infections, such as malaria and other viral exposures, which could drive polyclonal B-cell activation and more rapid antibody production. However, this heightened response does not necessarily translate into long-term immune durability, reinforcing the need for tailored vaccine regimens that account for regional immune kinetics. Moreover, the lack of correlation between neutralisation and binding antibodies in the Ugandan participants suggests potential qualitative differences in antibody function, warranting further investigation into isotype switching, avidity maturation, and alternative protective mechanisms beyond neutralisation.

### Study Limitations

While this study provides important insights, certain limitations must be acknowledged. The relatively small sample sizes may limit the generalisability, and future studies should validate these findings in larger, more diverse cohorts. Additionally, this study focused exclusively on humoral responses and did not assess T-cell immunity, which is a pivotal component of SARS-CoV-2 clearance and long-term protection [31,32,33]. Comprehensive profiling of cellular immunity is essential to elucidate the full spectrum of SARS-CoV-2 immunity. We aimed to compare responses across the populations in Uganda and the UK, but there was limited information on the baseline characteristics of the study participants, including race/ethnicity and comorbidities, all of which could be relevant to the observed immune response profiles. However, the observed demographic differences, such as age and gender [34], also varied slightly between cohorts, and may have influenced the results despite statistical adjustments. While statistical adjustments were applied, residual confounding may still influence the results. Finally, this study reflects early pandemic dynamics before the emergence of SARS-CoV-2 variants, highlighting the need for ongoing research to assess how these immune patterns adapt to evolving viral landscapes.

## 5. Conclusions

In conclusion, this study provides suggestive evidence of geographically distinct immune responses to SARS-CoV-2, underscoring the importance of population-specific approaches to vaccination. The pronounced early antibody response observed in Ugandan participants, coupled with its rapid decline, suggests that more frequent boosting may be necessary to sustain protective immunity in similar populations. Conversely, the UK cohort exhibited a more gradual but sustained antibody increase, implying robust immune memory formation. These findings provide a framework for refining vaccine strategies to optimise both immediate and long-term protection across diverse populations.

Ultimately, this study advances our understanding of how geographical and immunogenetic factors shape SARS-CoV-2 immunity, with direct implications for vaccine design, epidemic preparedness, and global health policies. Future studies should build upon these findings by integrating cellular immunity assessments, expanding cohort sizes, and exploring the impact of emerging variants to develop a holistic understanding of population-specific immune responses. By leveraging these insights, we can enhance the effectiveness of vaccines and immunological interventions, ensuring equitable protection against SARS-CoV-2 and future emerging infectious threats worldwide.

## Figures and Tables

**Table 1:** Baseline characteristics of the study population by nationality. Differences between groups were assessed using a chi-square test.

Characteristic		Total n (col %)	UG n (col %)	UK n (col %)	*p*-Value
Total		43 (100%)			
	UG	29 (67.4)			
	UK	14 (32.6)			
Sex					0.384
	Female	16 (37.2)	9 (31.0)	7 (50.0)	
	Male	27 (62.8)	20 (69.0)	7 (50.0)	
Age group ^§^					0.0811
	20–34	19 (51.4)	16 (55.2)	3 (37.5)	
	35–50	13 (35.1)	11 (37.9)	2 (25.0)	
	50+	5 (13.5)	2 (6.9)	3 (37.5)	
Admission symptoms *					0.0317
	No	28 (77.8)	14 (63.6)	14 (100.0)	
	Yes	8 (22.2)	8 (36.4)	0 (0.0)	

^§^ Six participants in the UK had missing age data. * Seven participants in the UG population had missing admission symptoms data. Symptoms reported included runny nose, fever, cough, headache, general weakness, blocked nostrils, and severe abdominal pain. The *p*-values indicated are from the χ^2^ test.

**Table 2:** Proportions of participants across neutralisation titre ranges in Uganda and the UK.

Neutralisation Range	Total n (%)	UG n (%)	UK n (%)
Total	127 (100)	61 (48.0)	66 (52.0)
>2000	13 (10.2)	10 (16.9)	3 (4.5)
500–2000	17 (13.4)	8 (13.1)	9 (13.6)
100–500	38 (29.9)	13 (21.3)	25 (37.9)
25–100	29 (22.8)	21 (34.4)	8 (12.1)
≤25	30 (23.6)	9 (14.8)	21 (31.8)

Table 2 presents the distribution of participants with varying neutralisation titre levels, stratified by location (UG and the UK). Neutralisation titres are categorised into the following five ranges: >2000, 500–2000, 100–500, 25–100, and <25. Each row displays the total number and percentage of participants in each titre range, with corresponding column percentages for UG and the UK.

## Data Availability

The raw data supporting the conclusions of this article will be made available by the authors upon request from the corresponding author (Jennifer Serwanga).
